# Effects of early exercise on cardiac function and lipid metabolism pathway in heart failure

**DOI:** 10.1111/jcmm.17908

**Published:** 2023-08-31

**Authors:** Sérgio Luiz Borges de Souza, Gustavo Augusto Ferreira Mota, Vitor Loureiro da Silva, Danielle Fernandes Vileigas, Paula Grippa Sant'Ana, Cristina Schmitt Gregolin, Rebeca Lopes Figueira, Sabrina Setembre Batah, Alexandre Todorovic Fabro, Gilson Masahiro Murata, Silmeia Garcia Zanati Bazan, Marina Politi Okoshi, Antonio Carlos Cicogna

**Affiliations:** ^1^ Department of Internal Medicine, Botucatu Medical School São Paulo State University Botucatu Brazil; ^2^ Department of Pathology and Legal Medicine, Ribeirão Preto Medical School University of São Paulo São Paulo Brazil; ^3^ Department of Internal Medicine, Faculty of Medicine University of São Paulo São Paulo Brazil

**Keywords:** angiogenesis, aortic stenosis, fatty acid oxidation, physical exercise, pressure overload, SIRT1

## Abstract

We employed an early training exercise program, immediately after recovery from surgery, and before severe cardiac hypertrophy, to study the underlying mechanism involved with the amelioration of cardiac dysfunction in aortic stenosis (AS) rats. As ET induces angiogenesis and oxygen support, we aimed to verify the effect of exercise on myocardial lipid metabolism disturbance. Wistar rats were divided into Sham, trained Sham (ShamT), AS and trained AS (AST). The exercise consisted of 5‐week sessions of treadmill running for 16 weeks. Statistical analysis was conducted by anova or Kruskal–Wallis test and Goodman test. A global correlation between variables was also performed using a two‐tailed Pearson's correlation test. AST rats displayed a higher functional capacity and a lower cardiac remodelling and dysfunction when compared to AS, as well as the myocardial capillary rarefaction was prevented. Regarding metabolic properties, immunoblotting and enzymatic assay raised beneficial effects of exercise on fatty acid transport and oxidation pathways. The correlation assessment indicated a positive correlation between variables of angiogenesis and FA utilisation, as well as between metabolism and echocardiographic parameters. In conclusion, early exercise improves exercise tolerance and attenuates cardiac structural and functional remodelling. In parallel, exercise attenuated myocardial capillary and lipid metabolism derangement in rats with aortic stenosis‐induced heart failure.

## 1 INTRODUCTION

Heart failure (HF) is a syndrome with an important morbidity and mortality impact worldwide and is an outcome of exposure of the heart to chronic stress, such as pressure overload (PO).^1^ Chronic cardiac overload is characterized by time‐dependent hypertrophy followed by diastole and systole decline, and subsequently, overt HF. The myocardial remodelling includes a set of changes including capillary rarefaction and oxygen deficit^2^; the imbalance between hypertrophy and angiogenesis, triggered by endothelial growth factor (VEGF) deficiency, limits the oxygen supply and leads to a disturbance of energy substrate metabolism and subsequently mechanical dysfunction.^3^

Fatty acid (FA) oxidation, the main source of energy generation by the healthy heart, is reduced during HF, accompanied by a broad downregulation of the FA metabolic pathway genes, as widely reported by studies in rodents^4^ and humans.^5^ Regarding PO, the downregulation of the peroxisome proliferator activated receptor‐α (PAPRα) is found to play an important role in decreased FA oxidation.^6^, ^7^ However, deficient lipid catabolism has also been found in parallel with unchanged^8^ or upregulated PPARα expression.^9^ In addition, recent studies with PO‐induced HF demonstrated that PPARα heterodimerizes with sirtuin 1 (SIRT1), and this complex (PPARα‐SIRT) suppresses the transcription of PPARα target genes, which are part of myocardial FA oxidation gear.^10^, ^11^

The high HF mortality rate despite the growing progress in the therapeutic makes the treatment of this syndrome an ongoing challenge.^1^ Aerobic exercise training (ET) as a non‐pharmacological method for the management of HF has been extensively described.^12^, ^13^ Studies that evaluated its influence on the transition to failure in a model of aortic stenosis (AS) showed significant attenuation of cardiac dysfunction,^14^, ^15^, ^16^ while other investigations using the same experimental model and ET protocol did not find similar results^17^, ^18^; the better results were found when exercise was implemented before severe cardiac dysfunction.

In line with the impact of energy perturbation on HF, exercise has been demonstrated to be a potential modulator of metabolic derangements that occur in the pathological heart^19^; adjustments in the FA capture and oxidation, in addition to improved mitochondrial biogenesis, have been shown in a few experimental assays with myocardial infarction and aortic insufficiency models.^19^, ^20^ The influence of early exercise on lipid metabolism of the failing heart undergoing severe myocardial hypertrophy of AS remains unknown so far.

Vast literature demonstrates that exercise improves VEGF signalling stimulating angiogenesis in a normal^21^, ^22^ or pathological condition,^23^, ^24^ especially in young animals.^25^ ET‐induced vascular capillarisation could attenuate myocardial oxygen shortage and consequently improve FA oxidation and energy production. Here we aimed to study the role of early exercise in generating cardioprotection and preserving the lipid metabolic pathway in aortic stenosis rats.

## 2 MATERIALS AND METHODS

### 2.1 Animals

Twenty‐one‐day‐old male *Wistar* rats weighing 70–90 g were purchased from the Central Animal House, Botucatu Medical School, Unesp. Rats were housed in collective polypropylene cages in a climate‐controlled environment at 23°C with a reverse 12:12‐h light/dark cycle and free access to food and water. All experiments and procedures were performed according to the Brazilian Guide for the Care and Use of Laboratory Animals and approved by Botucatu Medical School Animal Research Ethics Committee (protocol number 1239/2017).

### 2.2 Aortic stenosis procedure and experimental design

Supra‐valvar AS was surgically induced as described previously.^26^ Briefly, rats were anaesthetised and the heart was exposed via a median thoracotomy and a silver clip was placed on the ascending aorta. Sham animals underwent the same procedure but without the constriction of the aorta.

Initially, rats underwent either supra‐valvar aortic stenosis (AS) or Sham surgery (Sham). After 2 weeks, the animals were redistributed to be kept untrained (Sham, *n* = 16 and AS, *n* = 16) or submitted to exercise training (ShamT, *n* = 16 and AST, *n* = 14) for 16 weeks. The mortality rate did not differ between AS and AST. Two AS rats were removed from the study due to an unsatisfactory remodelling related to the clip placement in the aorta. Regarding the AST group, one rat had collateral circulation and two others were unable to adapt properly to the treadmill and were also excluded from the study.

Animals of all groups had the functional capacity and in vivo cardiac function evaluated before and after the exercise training period (i.e. 2 and 18 weeks after surgery, respectively), and subsequently, rats were submitted to euthanasia for additional analyses (Figure S1).

### 2.3 Treadmill exercise testing (TET)

Functional capacity was assessed before and after the ET period. TET was performed on a motorized treadmill for rats (AVS Projetos). Rats underwent 5 m/min/day test environment adaptation for 1 week. Maximum running speed and total time were recorded. Briefly, TET began at 6 m/min and increased by 3 m/min every 3 min until exhaustion. Exhaustion was defined as the non‐maintenance of the race at the proposed speed. The TET results were also used to prescribe the exercise program.

### 2.4 Aerobic exercise training

The ET protocol was modified from previous studies.^27^, ^28^ Rats were exercised for 16 weeks, 5 days a week at 60% of the maximal speed, achieved during the TET. The test was performed before the first week of training to initial exercise prescription and after the fourth, eighth and twelfth week to adjust running speed. Exercise duration from the first to the fourth week was progressively added 10 min per week until 60 min/day and then remained constant. During the training, animals received low‐voltage electrical stimulation.

### 2.5 Echocardiographic study

Measurements were performed after anaesthesia by intramuscular injection of a mixture of ketamine (50 mg/km) and xylazine (1 mg/kg), using an apparatus (Vivid S6, General Electric Medical Systems) equipped with a 5–11.5 MHz multifrequency probe, as recently described in detail.^29^

### 2.6 Anatomical data and heart failure features

The nutritional profile was evaluated according to body weight, fat deposits and food intake; body weight and food intake were measured weekly.

At euthanasia, was observed the occurrence of HF features in vivo (tachypnoea) and *post‐mortem* (ascites, pleural effusion, atrial thrombi and liver congestion) in the AS and AST groups. Two blind observers carried out a subjective evaluation of HF signals throughout the experiment as previously performed.^28^ Atria (AT) and left (LV) and right (RV) ventricles were dissected and weighed to determine cardiac hypertrophy. Tibia length was used to normalisation of cardiac parameters.

### 2.7 Immunohistochemistry

Slices of LV were kept in buffered formalin for 12 h at room temperature, followed by sequential dehydration in increasing concentrations of alcohol, cleared in xylene and embedded in paraffin. The blocks were cut into 3‐μm sections for immunohistochemical staining.

For immunohistochemistry, marked and pre‐assembled blades were dewaxed in xylene and rehydrated in decreasing concentrations of alcohol until water. For antigen recovery, the slides were heated at 98°C with citrate buffer (pH 6.0) for 30 min and then incubated in 0.3% hydrogen peroxide at room temperature for endogenous tissue peroxidase block. Then, they were washed with Tris‐buffered saline/Tween 20 (TBST), pH 7.5 and incubated with primary antibodies against VEGF (ab1316, Abcam; 1:200), VEGF receptor 1 (VEGFR1; 13,687‐1‐AP, Proteintech; 1:200) and 2 (VEGFR2; 9698 s, Cell Signalling; 1:200) and differentiation cluster 34 (CD34; ab81289, Abcam; 1:200) for 1 h in a humid chamber at room temperature. Slides were washed again with TBST, incubated with a secondary peroxidase horseradish polymer‐conjugated antibody for 30 min at room temperature, washed with TBST, incubated with 3,3′ diaminobenzidine stain for 5 min and counterstained with haematoxylin. Stereological point‐counting procedures were applied to perform a morphometric analysis.^30^

### 2.8 Protein expression by Western blot method

We analysed the expression of cardiac lipid metabolism proteins by the Western blot method as previously described in detail.^28^ As severe LV hypertrophy is related to the impaired angiogenesis pathway, we also studied the expression of key proteins that regulate angiogenesis and signal hypoxia. Primary antibodies: HIF1α (ab463, Abcam; 1:500); fatty acid transporter (CD36; ab133625, Abcam; 1:1000); carnitine palmitoyltransferase 1 (CPT1; NBP1‐59576, Novus Biological; 1:1000); fatty acid‐binding protein (FABP3; ab133585, Abcam; 1:1000); acyl‐CoA dehydrogenase (ACADL; ab196655, Abcam; 1:3000); medium‐chain acyl‐CoA dehydrogenase (MCAD; ab110296, Abcam; 1:1000); 2,4‐dienoyl‐CoA reductase (DECR1; ab198848, Abcam; 1:2000). AMP‐activated protein kinase (AMPK; 2532 s, Cell Signaling; 1:1000); phosphorylated AMPK (Thr 172) (pAMPK; 2535 s, Cell Signaling; 1:500); SIRT1 (8469 s, Cell Signaling; 1:1000); PPARα (ab8934, Abcam; 1:1000); retinoid X receptor (RXRα; 3085 s, Cell; 1:1000); peroxisome proliferator‐activated receptor gamma coactivator 1α (PGC1α; 20,658‐1‐AP, Proteintech; 1:1000). Targeted bands were normalized to the expression of cardiac GAPDH (sc‐32,233, Santa Cruz Biotechnology; 1:2000).

### 2.9 Metabolic enzymatic assays

Activities of the enzymes beta‐hydroxyacyl‐CoA dehydrogenase (OHADH), citrate synthase (CS) and creatine kinase (CK) that participate in fatty acid metabolism and citric acid cycle (CAC) were analysed in the myocardium. LV samples were homogenized 1:20 (wt/vol) in 50 mM Tris–HCl, 1 mM EDTA and protease inhibitor cocktail, pH 7.4, using a Bullet Blender storm, (Next Advanced). The lysate was centrifuged at 12000 *g* for 10 min at 4°C and the supernatant was collected. All enzyme activities were determined at 25°C using a Spectra Max 250 microplate spectrophotometer (Molecular Devices), and the assay buffer without the sample was used as blank. CK was assayed in a medium consisting of 91.1 mM Tris–HCL, 1 mM ATP, 0.262 mM ß‐NAD reduced form, 0.728 mM PEP, 10 mM creatine, 5 mM KCl, 2 mM MgSO_4_, 10 units lactic dehydrogenase, 0.4–0.6 unit pyruvate kinase and 0.05% Triton X‐100. pH 7.4. The total assay volume was 165 and 2 microliter of homogenate was added. The assay was initiated by the addition of creatine. The absorbance of CS and CK assays was monitored at 412 nm. For OHADH, the reaction contained 100 mM PBS pH 7.3, 0.45 mM NADH and 1.0 mM acetoacetyl CoA. CS activity was measured in a reaction containing 100 mM Tris–HCl, 1 mM MgCl_2_, 1 mM EDTA, 0.2 mM dithio‐bis (2‐nitrobenzoic acid), 0.3 mM acetyl CoA and 0.5 mM oxaloacetate, pH 8.2. The rate of change in absorbance was monitored at 340 nm (*ɛ* = 13.6 μmol mL‐_1_).

### 2.10 Adenosine triphosphate content

ATP content was measured as previously.^31^ Briefly, LV was homogenized in perchloric acid (0.6 N) and the supernatant was neutralized using potassium bicarbonate aqueous solution (1 M). The assay medium consisted of 75 mM Tris–HCl, 7.5 mM MgCl_2_, 0.8 mM EDTA, 1.5 mM KCl, 4 mM 2 mercaptoethanol, 0.4 mM NADP^+^, 1 m mM glucose, 1.4 units glucose‐6‐phosphate dehydrogenase, 0.05% Triton X‐100, pH 7.2. The total assay volume was 165 μL and 10 μL of fresh homogenate was added. The assay was initiated by the addition of glucose and monitored at 340 nm.

### 2.11 Statistical analysis

Data are expressed as mean ± standard deviation or median and percentiles. Kolmogorov–Smirnov normality test was used to test data normal distribution. Two‐way analysis of variance (anova) followed by Bonferroni *post‐hoc* test (parametric distribution) or Kruskal–Wallis followed by Dunn's (non‐parametrical distribution) was used to analyse differences between groups. Goodman test was used to test differences in HF features. The correlation matrix was generated using a two‐tailed Pearson's correlation test.^32^ All analyses were performed using Sigma Plot 12.0 (Systat Software, Inc.), except for the correlation matrix that was carried out in GraphPad Prism 8 (GraphPad Software Inc.). The significance level was set at 5%.

## 3 RESULTS

### 3.1 Nutritional parameters

Nutritional data are shown in Table S1. Body weight of ShamT and AS groups were lower than Sham, while AST presented lower body weight than AS. Except for epididymal, all fat deposits were reduced in aortic stenosis rats regardless of exercise training. Additionally, food ingestion was reduced in AS rats, and this scenario was attenuated by the exercise.

### 3.2 Functional capacity and heart failure features

**FIGURE 1:**
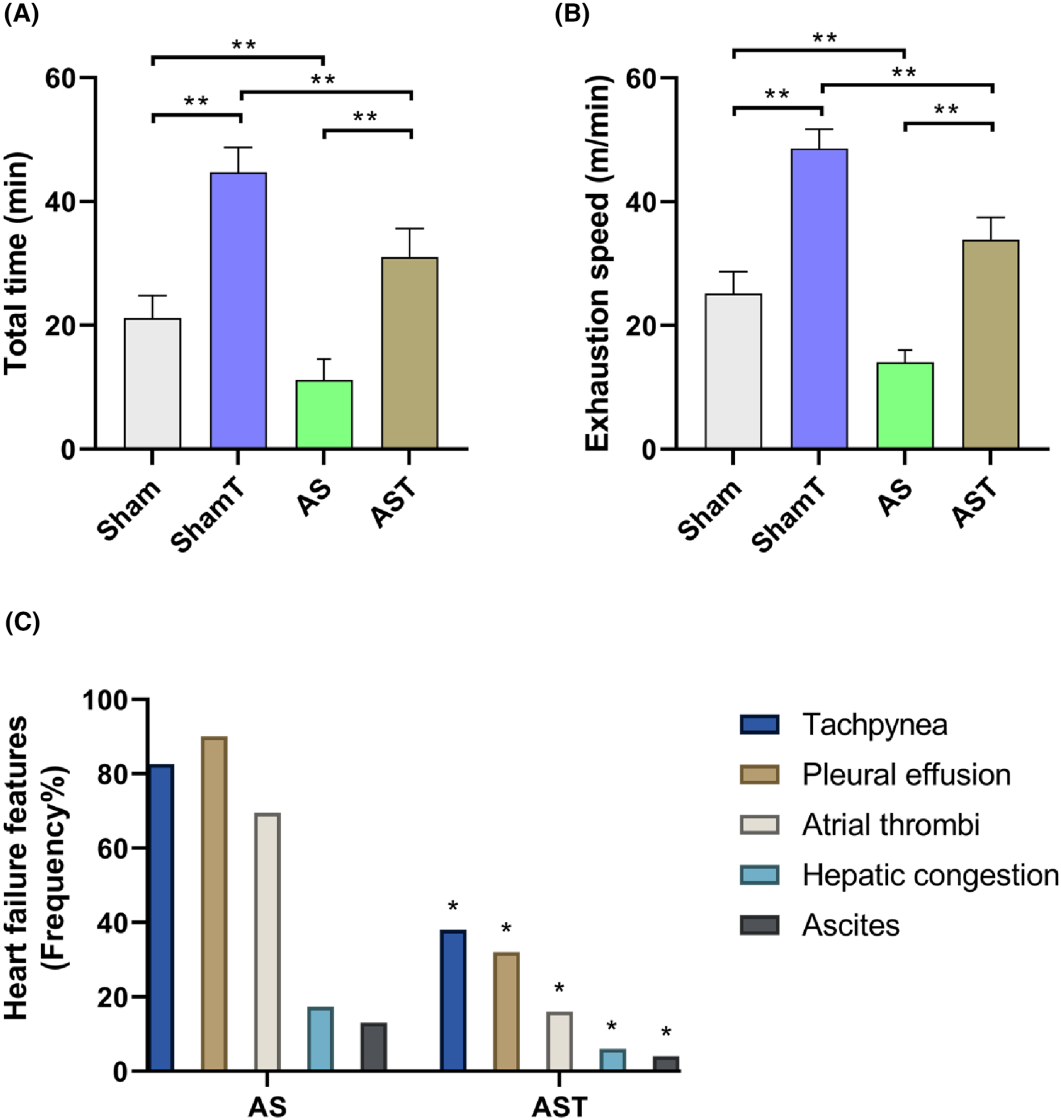
Final treadmill exercise testing. AS, untrained aortic stenosis; AST, trained aortic stenosis; ShamT, trained Sham. Data are mean ± SD or relative frequency; anova and Goodman; *p < 0.05. **p < 0.001. (*n* = 14–16 each group).

The final test showed that stenosis impaired exercise tolerance while exercise improved it in both normal and pathological conditions. Symptoms related to HF were observed in all untrained animals while the exercised group had these features occurrence substantially reduced (Figure 1).

### 3.3 Cardiac anatomical profile

Cardiac anatomical parameters are presented in Table S2. Atria, RV, and LV weights were greater in AS group than in Sham, both in absolute and normalized to the tibia, indicating cardiac hypertrophy. Compared to AS, the AST group displayed a lower weight in all parameters.

### 3.4 Echocardiographic evaluation

The initial echocardiogram was performed to ensure homogeneity between groups before exercise program (Table S3). Final echocardiographic assessment (18th‐week post‐surgery), is presented in Table 1. AS groups displayed LV hypertrophy, visualized by higher LV diastolic (LVDD) and systolic diameter (LVSD), and relative wall thickness (RWT), accompanied by LA enlargement. Additionally, stenosis induced systolic dysfunction characterized by lower mesocardial (MFS) and endocardial (EFS) fractional shortening, posterior wall thickness systolic velocity (PWSV), Tei index and tissue Doppler image (TDI) of the systolic velocity of mitral annulus (TDI S′). Furthermore, diastolic dysfunction was also diagnosed by lower isovolumetric relaxation time and E‐wage deceleration time, and by the higher following ratios: E‐wave (E) to A‐wave, E to TDI early (E') velocity of mitral annulus and TDI E' to TDI late velocity of mitral annulus. However, the AST group in comparison to AST displayed an attenuated status of structural remodelling, characterized by reduced LVDD, LVSD, PWDT/Tibia and LA/AO ratio and cardiac dysfunction as visualized in systolic (MFS, ESF, PWSV and TDI S′) and diastolic (IVRT, EDT, and E/A), parameters. Heart rate values were similar between groups.

**TABLE 1:** Final echocardiographic data.

	Sham	ShamT	AS	AST
HR (bpm)	292 ± 27.1	317 ± 35.9	318 ± 52.0	323 ± 52.9
LVDD (mm)	7.44 ± 0.50	7.42 ± 0.51	8.42 ± 0.59^a^	7.56 ± 0.95^c^
LVDD/Tibia	1.69 ± 0.12	1.74 ± 0.12	1.97 ± 0.12^a^	1.74 ± 0.24^c^
LVSD (mm)	2.94 (2.81–3.32)	3.07 (2.81–3.32)	4.09 (4.09–4.60)^a^	3.41 (3.01–3.90)^c^
PWDT (mm)	1.53 (1.53–1.53)	1.53 (1.51–1.58)	2.81 (2.69–2.92)^a^	2.50 (2.30–2.62)^b^
PWDT/Tibia	0.35 ± 0.01	0.37 ± 0.06	0.66 ± 0.04^a^	0.60 ± 0.07^bc^
AO (mm)	3.83 (3.64–4.09)	3.96 (3.83–4.09)	4.09 (3.90–4.31)	4.09 (3.83–4.34)
LA (mm)	4.85 (4.60–5.05)	4.85 (4.60–5.11)	8.05 (7.73–8.63)^a^	7.15 (6.39–8.05)^b^
LA/AO (mm/g)	1.23 ± 0.10	1.27 ± 0.12	2.00 ± 0.18^a^	1.78 ± 0.32^bc^
RWT	0.41 (0.40–0.44)	0.41 (0.39–0.44)	0.69 (0.63–0.72)^a^	0.68 (0.57–0.78)^b^
MFS (%)	27.1 ± 3,78	27.5 ± 3.37	21.2 ± 4.16^a^	24.1 ± 2.76^bc^
EFS (%)	59.7 ± 3.07	59.4 ± 4.05	47.9 ± 5.73^a^	54.5 ± 5.21^bc^
PWSV (mm/s)	73.5 ± 5.33	70.8 ± 8.77	44.8 ± 6.48^a^	56.4 ± 11.4^bc^
Tei index	0.21 ± 0.08	0.26 ± 0.09	0.34 ± 0.16^a^	0.26 ± 0.11
TDI S (average, cm/s)	5.74 ± 0.33	5.73 ± 0.23	4.18 ± 0.50^a^	4.78 ± 0.43^bc^
IVRT (ms)	23.5 ± 2.31	22.9 ± 2.69	13.3 ± 2.69^a^	17.8 ± 4.58^bc^
EDT (ms)	48.6 ± 2.71	47.7 ± 3.93	25.9 ± 6.30^a^	37.4 ± 10.5^bc^
Mitral E (cm/s)	83.2 (82–87.0)	81.4 (78.6–91.1)	133 (116–143)^a^	104 (93.0–118)^b^
Mitral A (cm/s)	50.3 (48.2–53.6)	55.4 (47.1–62.7)	23.1 (20.3–26.6)^a^	34.8 (19.7–64.5)^b^
E/A (cm/s)	1.60 ± 0.18	1.83 ± 1.34	5.63 ± 0.96^a^	3.88 ± 2.48^bc^
TDI E' (average, cm/s)	5.65 ± 0.69	6.12 ± 0.71	5.11 ± 0.69^a^	5.52 ± 0.71^c^
TDI A' (average, cm/s)	3.84 ± 0.67	3.99 ± 0.37	2.78 ± 0.81^a^	3.51 ± 0.96^*p* = 0.07^
E/E' (cm/s)	15.3 (14.3–16.4)	14.0 (12.4–15.3)	24.8 (22.6–29.4)^a^	19.5 (16.6–23.0)^b^
E'/A' (cm/s)	1.47 (1.40–1.55)	1.54 (1.44–1.59)	1.98 (1.73–2.13)^a^	1.64 (1.53–1.95)^b^

*Note*: Data are mean ± SD or median and interquartiles.

Abbreviations: AO, aorta; AS, untrained aortic stenosis; AST, trained aortic stenosis; E/A, ratio between early (E) to late (A) diastolic mitral inflow; EDT, E‐wave deceleration time; EFS, endocardial fractional shortening; HR, heart rate; IVRT, isovolumetric relaxation time; LA, left atrial diameter; LVDD and LVSD, left ventricular (LV) diastolic and systolic diameters; MFS, mesocardial fractional shortening; PWDT, posterior wall diastolic thickness; PWSV, posterior wall shortening velocity; RWT, relative wall thickness; ShamT, trained Sham; TDI E' and A', TDI of early (E') and late (A') diastolic velocity of mitral annulus; TDI S, tissue Doppler imaging (TDI) systolic velocity of mitral annulus; Tei index, myocardial performance index.

*p* < 0.05. ^a^versus Sham; ^b^versus ShamT; ^c^versus AS. (*n* = 14–16 each group).

### 3.5 Angiogenesis study

**FIGURE 2:**
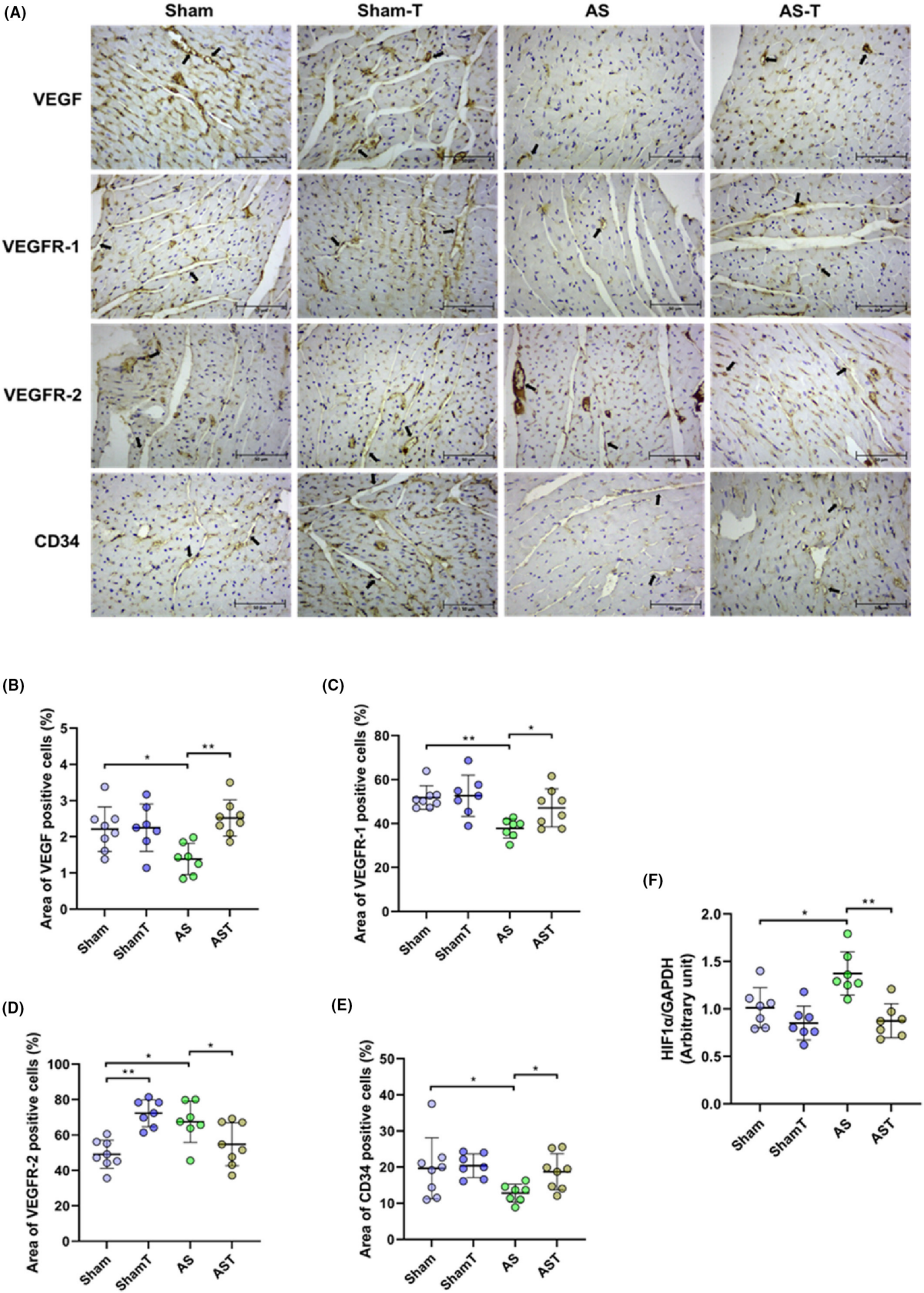
(A–E) Representative photographs of immunohistochemistry staining (A) and quantitation of the expression level of the VEGF, vascular endothelial growth factor (B); VEGFR‐1, VEGF receptor type 1 (C) and 2 (D); and CD34, differentiation cluster 34 (E) in myocardium sections (indicated by black arrows). Scale bar, 50 μm. (F) Quantification of the protein expression of hypoxia‐inducible factor 1‐α. AS, untrained aortic stenosis; AS, untrained aortic stenosis; AST, trained aortic stenosis; ShamT, trained Sham. Data are mean ± SD; anova; *p < 0.05. **p < 0.001. (*n* = 6–8 each group).

The effects of PO and exercise on cardiac angiogenesis are shown in Figure 2. VEGF expression was downregulated in AS group while it was maintained unaffected in AST. Moreover, VEGFR‐1 expression was also impaired by AS surgery and preserved by ET. Interestingly, the VEGFR‐2 level was upregulated in AS group relative to the Sham group and downregulated in AST relative to ShamT and AS groups. It was found that density of CD34 expression, a tissue vessel density marker, was reduced after PO and unaffected in AST hearts. HIF1α, a hypoxia marker, was found to be overexpressed in AS rats, and not in AST rats; this result corroborates the higher capillary density visualized in the immunohistochemical assay and, together, data may indicate a lower oxygen deficit in AST hearts.

### 3.6 Analysis of the fatty acid oxidation pathway properties

**FIGURE 3:**
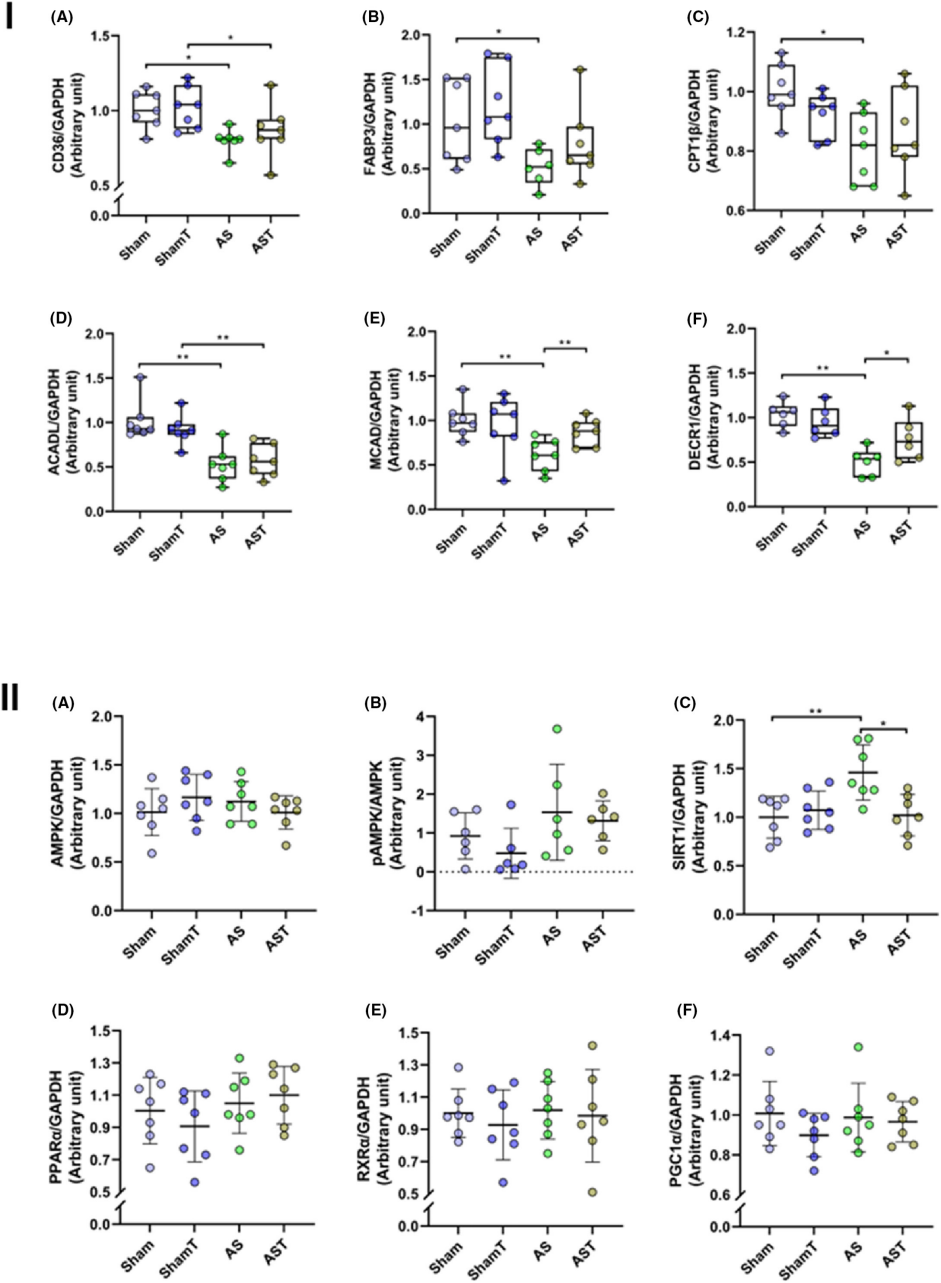
(I) Quantification of the expression levels of proteins involved with cardiac fatty acids translocation and intracellular transport, and β‐oxidation. CD36, fatty acid (FA) translocase (A); FA‐binding protein, FABP3 (B); carnitine palmitoyltransferase 1, CPT1β (C); acetyl‐CoA dehydrogenase, ACADL (D); medium‐chain acyl‐CoA dehydrogenase, MCAD (E); 2,4‐dienoyl‐CoA reductase, DECR1 (F). (II) Quantification of the expression levels of metabolic sensors and transcriptional effectors. AMP‐activated protein kinase, AMPK (A); phosphorylated AMPK, pAMPK (B); sirtuin 1, SIRT1 (C); peroxisome proliferator activated receptor‐α, PPARα (D); receptor X retinoid, RXR (E); peroxisome proliferator‐activated receptor gamma coactivator 1α, PGC1α (F). AS, untrained aortic stenosis; AST, trained aortic stenosis; ShamT, trained Sham. Data are mean ± SD; Kruskal–Wallis and anova; *p < 0.05. **p < 0.001. (*n* = 6–8 each group).

The expression of a set of proteins involved in lipid uptake, translocation and oxidation was evaluated and is shown in Figure 3I. AS group presented lower levels of CD36, CPT1β and FABP3 compared to Sham; AST rats, however, had unchanged CD36 expression. ET did not affect proteins of the intracellular and mitochondrial transport of FA when compared with AS group (Figure 3I,A–C). We also evaluated the expression of enzymes that catalyse the steps of β‐oxidation of saturate and unsaturated FA. ACADL, involved with the oxidation of unsaturated FA was markedly diminished in aortic stenosis rats regardless of exercise. MCAD and DECR1, which act on the oxidation of medium‐chain and polyunsaturated fatty acids, respectively, had their levels reduced by stenosis and recovered in AST rats, indicating a favourable impact of exercise in preserving myocardial mitochondrial β‐oxidation (Figure 3I,D–F). As impaired β‐oxidation contributes to energy imbalance status, we measured the metabolic sensors AMPK and SIRT1 (Figure 3II,A–C); only SIRT1 was overexpressed in AS group. Lastly, we analysed the expression of proteins related to the transcription of genes linked to FA transport and oxidation. Interestingly, the downregulation of PPARα proposed as the mechanism to reduce fatty acid oxidation (FAO) in hypertrophied hearts did not occur in our investigation; the protein level of the transcriptional effectors RXRα and PGC1α also remained unaffected in AS groups (Figure 3II,D–F).

### 3.7 Metabolic enzyme activity

**FIGURE 4:**
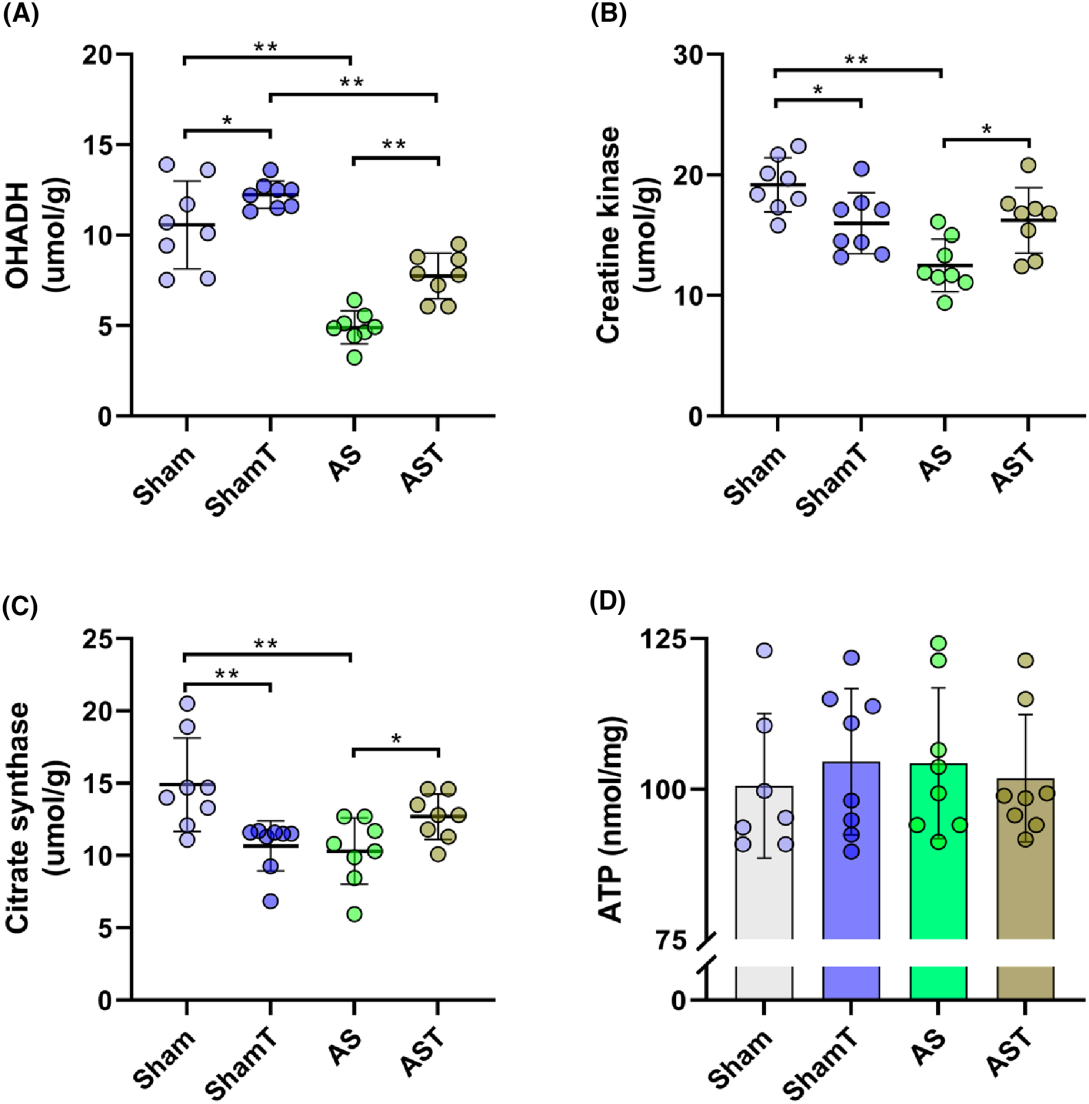
AS, untrained aortic stenosis; AST, trained aortic stenosis; ATP, adenosine triphosphate; OHADH, beta‐hydroxyacyl‐CoA dehydrogenase; ShamT, trained Sham. Data are mean ± SD; anova; *p < 0.05. **p < 0.001. (*n* = 08 each group).

A significant reduction was observed in the activity of OHADH, CS, and CK of AS hearts (Figure 4A–C). The AST group, however, presented all enzyme activities normalized at the level of their control and differed significantly from the AS group; interestingly, creatine kinase and citrate synthase activities were found reduced in the ShamT. Myocardial ATP content did not differ between groups (Figure 4D).

### 3.8 Global correlation map

**FIGURE 5:**
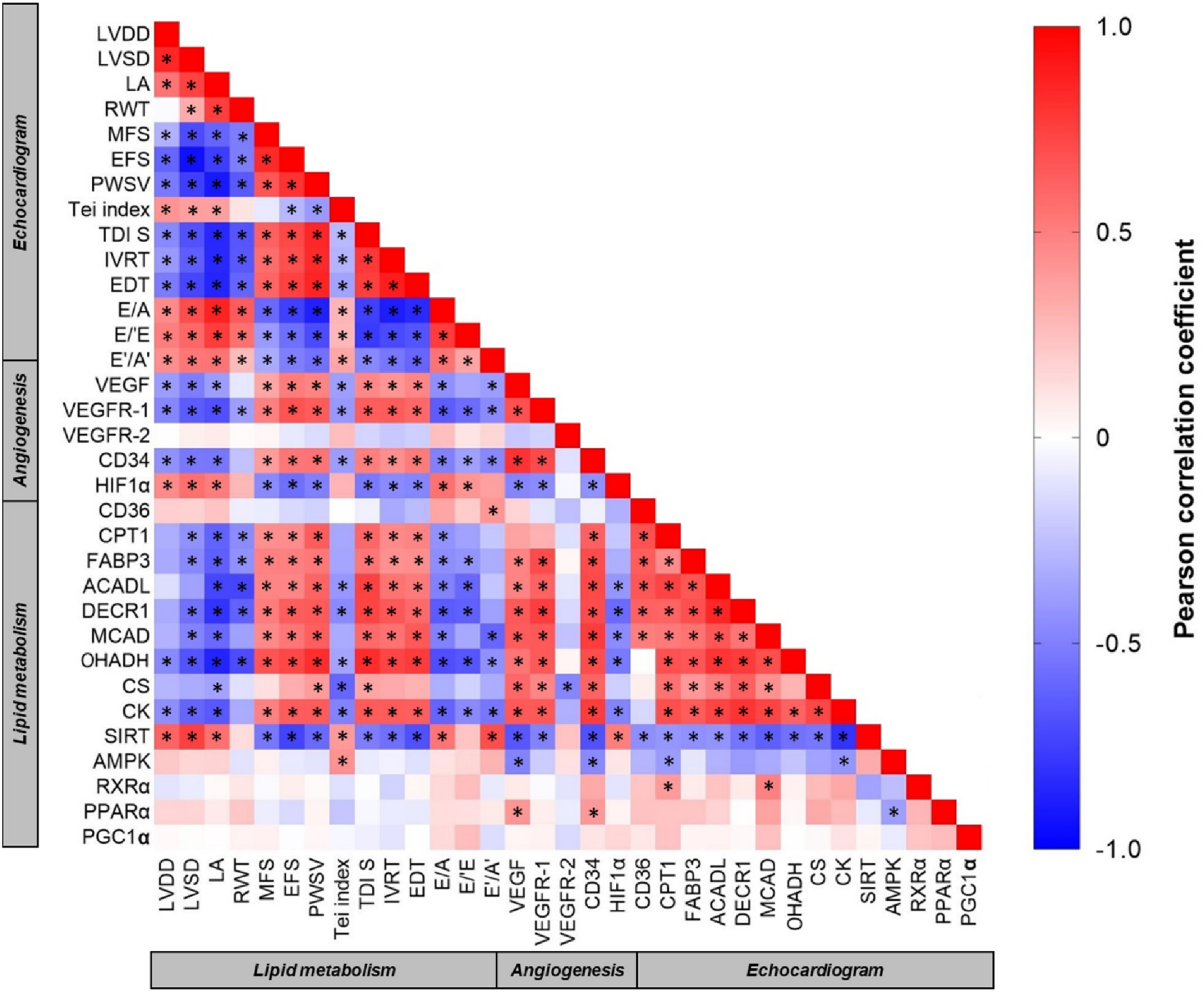
Global correlation map of angiogenesis, lipid energy metabolism, and cardiac function. Pairwise correlation of all parameters, resulting in a matrix of correlation coefficients where each variable is compared to all others. Red squares represent variables with a positive correlation with each other. The negative correlation is indicated in blue squares. ACADL, acyl‐CoA dehydrogenase; AKT, protein kinase B; AMPK, AMP‐activated protein kinase; CD34, differentiation cluster 34; CD36, fatty acid (FA) translocase; CK, creatine kinase; CPT1, carnitine palmitoyltransferase; CS, citrate synthase; DECR1, 2,4‐dienoyl‐CoA reductase; E'/A', ratio between E' to TDI of late diastolic velocity of mitral annulus; E/A, ratio between early (E) to late (A) diastolic mitral inflow; E/E', ratio between E and TDI of early diastolic velocity of mitral annulus (E'); EDT, E‐wave deceleration time; EFS, endocardial fractional shortening; FABP3, FA‐binding protein; HIF1α; hypoxia inducible factor 1‐α; IVRT, isovolumetric relaxation time; LA, left atrium; LVDD and LVSD, left ventricular (LV) diastolic and systolic diameters; MCAD, medium‐chain acyl‐CoA dehydrogenase; MFS, mesocardial fractional shortening; OHADH, beta‐hydroxyacyl‐CoA dehydrogenase; p53, p53 tumour suppressor protein; PGC1α, peroxisome proliferator‐activated receptor gamma coactivator 1α; PI3K, phosphoinositol 3 kinase; PPARα, peroxisome proliferator activated receptor‐α; PWSV, posterior wall shortening velocity; RWT, relative wall thickness; RXRα, receptor X retinoid‐α; SIRT1, sirtuin 1; TDI S, tissue doppler imaging (TDI) systolic velocity of mitral annulus; Tei index, myocardial performance index; VEGF, vascular endothelial growth factor; VEGFR1 and VEGFR2, VEGF receptor type 1 and 2. The data were analysed by Graphpad Prism 8 using two‐tailed Pearson's correlation test. Symbol (*) indicates a statistically significant correlation (*p* < 0.05).

Figure 5 displays the major association results in the format of a heatmap.

As expected, angiogenesis was positively correlated with metabolic parameters; VEGF with *PPARα, FABP3, ACADL, MCAD, DECR1, OHADH, CK* and *CS*; CD34 with *FABP3, CPT1β*, *ACADL, MCAD, DECR1* and *OHADH*. We also found a negative correlation between angiogenic markers (VEGF, CD34) and sensors of the cellular metabolic stress (AMPK, SIRT1). In addition, HIF1α was found negatively correlated with the lipid metabolism pathway (ACADL, DECR1, MCAD, OHADH and CK), which suggests a high relationship between hypoxia and energy perturbation during HF.

Heatmap also showed a positive correlation of proteins linked to FA transport and oxidation (CPT1β, FABP3, ACADL, DECR1, MCAD and OHADH) with systole (MFS, EFS, PWSV and TDI S′). Conversely, metabolism was inversely correlated with cardiac structural and diastole, except by SIRT1 which showed a positive correlation with hypertrophy and diastolic dysfunction indicators. Based on these results, lipid energy metabolism is primarily linked to systolic performance, while FAO decline occurs in parallel with pathological hypertrophy and diastolic dysfunction.

## 4 DISCUSSION

Searching for the therapeutic approaches for the management of heart diseases remains a challenge and non‐pharmacological strategies are emerging as an effective tools for prevention and treatment. Here, we showed that (1) early exercise training had a protective effect against AS‐induced pathological remodelling and cardiac dysfunction in rats, (2) corrected vascular rarefaction in the myocardium and (3) re‐established expression and activity of components of the myocardial lipid metabolism pathway during HF. These benefits were accompanied by a significant alleviation of the HF profile including functional capacity maintenance.

For this report, we adapted a training protocol from previous studies that found benefits in functional capacity and cardiac function with different experimental models of cardiac disease.^28^, ^29^, ^33^ Both trained groups presented higher functional capacity than untrained Sham and AS groups, as shown by others.^14^, ^17^

Previous studies attempt to rescue deficient cardiac function in aortic stenosis rats using a late exercise program that resulted in inconsistent conclusions.^16^, ^18^, ^28^ These studies started the training after the worsening of the cardiac disturbance, and different degrees of systolic disability appear to be linked to controversial results. Thus, the timing of the ET after aortic stenosis has not yet been optimized. As in sustained PO the remodelling becomes maladaptive early on^34^ leading to incompetence of adaptive molecular signalling pathways,^2^ for this study, we started the ET program before severe cardiac hypertrophy and dysfunction took place. The assumption that early ET could alleviate the dysfunctional scenario of cardiac overload raises from the recognized potential of its physiological signalling, in counteracting the pathological signalling cascades involved with heart remodelling and failure.^22^

Regarding cardiac structure, our data demonstrate that AST rats displayed alleviation of the hypertrophic process as visualized in the weight of chambers and echocardiogram parameters. Following the effect on cardiac structure, both diastolic, and systolic impairment were significantly attenuated by ET. Additionally, exercise reduced the occurrence of tachypnoea, pleural effusion, atrial thrombi, liver congestion and ascites, classical symptoms of HF observed in experimental trials^28^, ^33^; ET also prevented the decline in the food consumption in AS rats. Collectively, the outcomes indicate that early ET can alleviate stenosis‐induced HF and that early aerobic exercise is safe in young AS rats. An intriguing result was the reduction in citrate synthase activity in the ShamT group. However, while increased CS activity is considered a marker of exercise training efficiency, its significance in normal rats remains controversial,^35^, ^36^, ^37^ and similar results have been found by other researchers.^38^

We mainly aimed to investigate the underlying mechanisms involved with the benefits of exercise on the failing heart. Several studies have documented that transition from hypertrophy towards HF is associated with myocardial capillary rarefaction,^39^, ^40^, ^41^ which becomes unable to support oxygen and nutrient demands imposed by chronic PO.^42^ We presently hypothesized that early exercise would lead to the reactivation of the angiogenesis. As expected, AS rats presented a significant reduction of myocardial capillary density assessed by CD34 expression, which was associated with impaired expression of VEGF, as largely reported.^42^, ^43^ In contrast, exercise prevented capillary rarefaction after stenosis by maintaining a normal expression of VEGF and its receptors. Our findings corroborate lines of evidence showing that ET induces a myocardial angiogenic profile characterized by activation of the VEGF pathway in several experimental models^23^, ^44^, ^45^, ^46^ and improve the knowledge about the angiogenic scenario in failing AS heart.

Our data reveal that the preservation of VEGF levels and capillary density in hearts of AST occurred in parallel with reduced HIF1‐α protein content compared to the untrained AS group, which presented HIF1‐α overexpression. A recent study conducted in our laboratory found a time‐dependent increase in HIF1‐α levels during the worsening of cardiac function.^47^ These results strengthen the assumption that early exercise, by angiogenesis supporting, can mitigate the PO‐induced myocardial hypoxia, as the present data of normalized HIF1‐α expression points out.

Based on the above findings related to angiogenesis and hypoxia, we tested whether myocardial lipid metabolism is also associated with cardioprotection promoted by early exercise. This approach is essential since increasing evidence demonstrates that energy metabolic derangements contribute to the pathogenesis of cardiac functional decompensation,^11^ and that impaired FAO precedes the onset of the PO‐induced HF.^48^

To the best of our knowledge, we provide the first scientific literature about the effects of early ET on cardiac lipid metabolism in AS‐induced HF. Pathological hypertrophy was accompanied by a disturbance in the FA utilisation pathway; AS rats presented a reduced expression of key components involved with fatty acid transport and β‐oxidation. Conversely, the impact of PO on the FA pathway was less important in the exercised group, as visualized in the results session, which suggests that ET in course of HF attenuates the pathological pattern of energy generation from FA. In line with present data, the downregulation of genes involved in FA metabolism, from uptake to cleavage within the mitochondrial β‐oxidation cycle, is widely shown.^49^, ^50^, ^51^ However, although studies conducted in rats revealed that treadmill running triggered a distinct cardio‐metabolic gene profile compared to PO or myocardial infarction,^52^, ^53^ the paucity of data studying the influence of exercise on the modulation of myocardial metabolism in the AS failing heart does not allow us to compare our results. Nevertheless, the metabolic coordination induced by ET has been also shown by experimental studies with myocardial infarction,^20^, ^54^ aortic regurgitation^55^ and spontaneously hypertensive rats.^56^

The mechanisms associated with the decreased FAO pathway appear to be related to a transcriptional disorder that includes transcription factors, especially PPARα.^11^, ^57^ PPARα heterodimerizes with RXRα and recruits PGC1α to promoving transcription of PPARα targets involved with lipid metabolism, including FA uptake, transport and β‐oxidation.^58^ It has been described that decreased expression of PPARα enrols part in the downregulation of genes of FA metabolism; however, its decreased expression is not uniformly reported in PO‐induced HF.^11^ In the present study, PPARα protein level was similar between groups, as well as RXRα and PGC1α. Our data even indicate that concomitant maintenance of PPARα and RXRα in the AS group did not prevent damage in the cardiac FAO pathway, as described by others,^8^, ^57^ suggesting that protein level of PPARα per se is not the only factor that leads to metabolic imbalance during HF. Noteworthy, downregulation of the lipid metabolism has been linked to a mechanism related to an atypical interaction between PPARα and Sirt1^10^, ^11^; Sirt1 overexpression favours the increased formation of PPARα‐Sirt1 complexes over PPARα‐RXRα, which inhibits the transcription of some target genes involved in FAO.^10^ As Sirt1 was not overexpressed in the AST group, PPARα‐RXRα activity was likely unimpaired, responding, at least in part, by the restored FAO protein levels. Thus, ET might be involved with the balancing of transcriptional regulation of FAO signalling genes throughout pathological heart remodelling in aortic stenosis.

Loss of ATP and phosphocreatine are classical characteristics of the failing heart.^59^ In our experiment the myocardial ATP pool did not differ between groups, which would be expected due to important cardiac dysfunction and energy metabolism stress. On the other hand, the activity of CK was significantly reduced in AS group, which can bring down the phosphocreatine supply and consequently increase the free adenosine diphosphate (ADP), triggering inhibition of a set of intracellular enzymes, including that of the contractile mechanism.^60^ Noteworthy, attenuated cardiac dysfunction in the AST group occurred together with CK activity maintenance.

**FIGURE 6:**
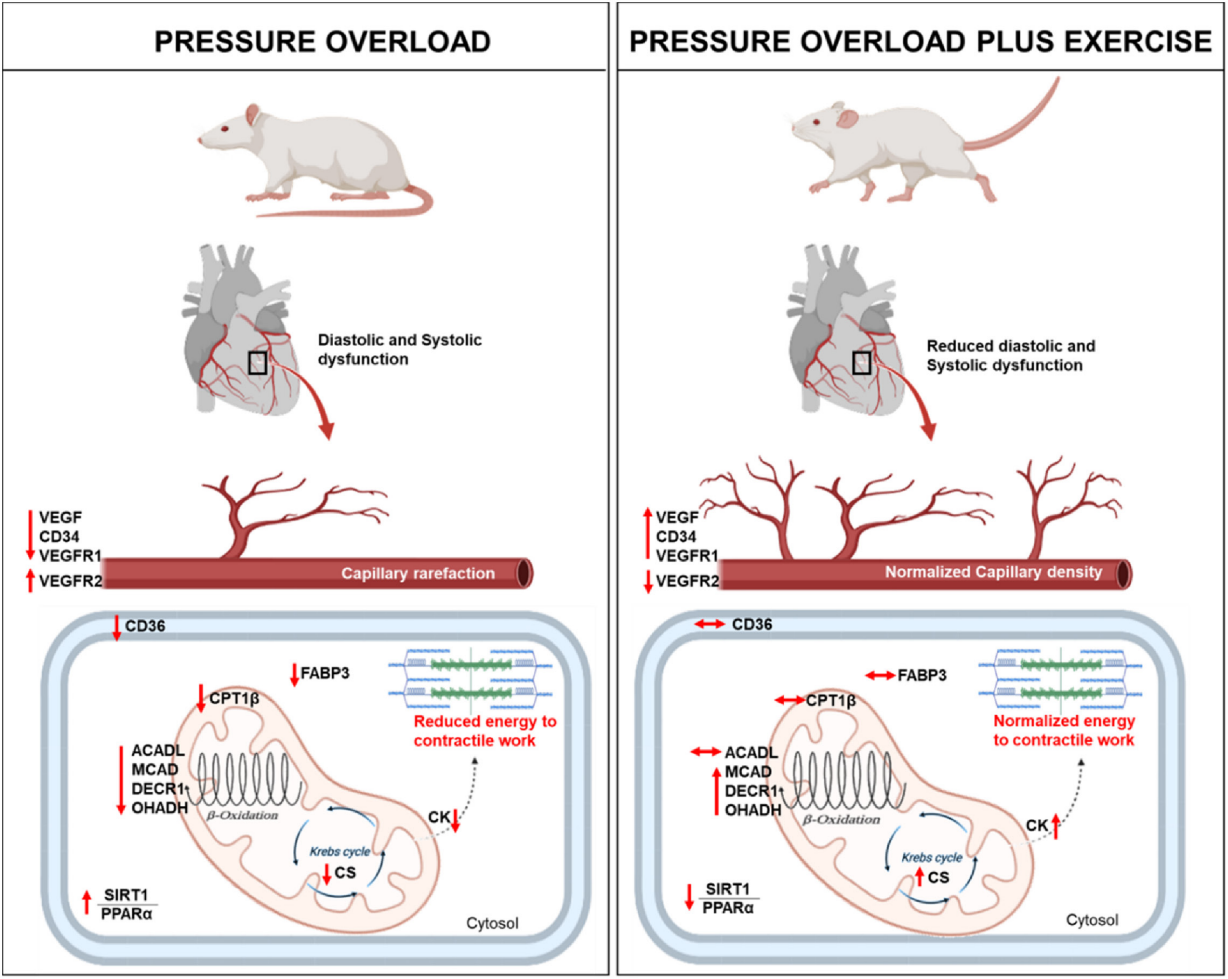
Overview of the effects of pressure overload (PO) and PO plus exercise on cardiac function, angiogenesis and lipid energy metabolism. ACADL, acyl‐CoA dehydrogenase; CD34, differentiation cluster 34; CD36, fatty acid (FA) translocase; CK, creatine kinase; CPT1, carnitine palmitoyltransferase; CS, citrate syinthase; DECR1, 2,4‐dienoyl‐CoA reductase; FABP3, FA‐binding protein; MCAD, medium‐chain acyl‐CoA dehydrogenase; OHADH, beta‐hydroxyacyl‐CoA dehydrogenase; PPARα, peroxisome proliferator activated receptor‐α; SIRT1, sirtuin 1; VEGF, vascular endothelial growth factor; VEGFR‐1 and VEGFR2, VEGF receptor type 1 and 2.

It was demonstrated a positive correlation between angiogenesis and FA signalling, as well as a negative correlation between cardiac remodelling with both angiogenesis and metabolism, endorsing our results. In summary, our data suggest that early exercise rescues capillary density and attenuates the disturbance of the FAO pathway together with amelioration of cardiac performance, even in the setting of severe hypertrophy. An overview of the results are presented in the Figure 6.

## 5 LIMITATIONS

In our study, we provide an initial analysis of the lipid metabolism since the static measurement of enzymes does not specifically indicate a change in fatty acid oxidation. Additional studies will be needed to deepen the knowledge of the substrate flux and tissue oxygen availability of the hypertrophied heart under early exercise. Also, we did not study the interaction between SIRT1 and RXR, therefore, our data are limited to supposing a plausible mechanism. Regarding energy production, we did not measure the phosphocreatine content, which is found to be initially reduced in heart failure and could reveal energy depletion.

## 6 CONCLUSIONS

In conclusion, early exercise attenuated cardiac remodelling, reduced HF symptoms and improved the functional capacity of aortic stenosis rats. For these results, we demonstrate that early exercise triggers a consistent effect on the management of AS‐induced HF. Regarding the underlying mechanisms involved with the beneficial effects of ET in AS rats, we raise the assumption that attenuation of cardiac dysfunction potentially is linked to the preservation of lipid energy metabolism, which in turn is related to angiogenesis restoration.

## AUTHOR CONTRIBUTIONS

**Sérgio Luiz Borges de Souza:** Conceptualization (equal); data curation (equal); formal analysis (equal); funding acquisition (equal); investigation (equal); methodology (equal); project administration (equal); resources (equal); software (equal); supervision (equal); validation (equal); visualization (equal); writing – original draft (equal); writing – review and editing (equal). **Gustavo Augusto Ferreira Mota:** Conceptualization (equal); investigation (equal); methodology (equal); resources (equal); supervision (equal); visualization (equal). **Vitor Loureiro da Silva:** Conceptualization (equal); investigation (equal); methodology (equal). **Danielle Fernandes Vileigas:** Methodology (equal). **Paula Grippa Sant'Ana:** Methodology (equal). **Cristina Schmitt Gregolin:** Methodology (equal). **Rebeca Lopes Figueira:** Methodology (equal). **Sabrina Setembre Batah:** Methodology (equal). **Gilson Masahiro Murata:** Conceptualization (equal); investigation (equal); resources (equal); supervision (equal). **Silmeia Bazan:** Methodology (equal). **Alexandre Fabro:** Conceptualization (equal); resources (equal); supervision (equal). **Marina Politi Okoshi:** Resources (equal); visualization (equal). **Antonio Carlos Cicogna:** Conceptualization (equal); data curation (equal); formal analysis (equal); funding acquisition (equal); project administration (equal); supervision (equal); validation (equal); visualization (equal); writing – original draft (equal); writing – review and editing (equal).

## CONFLICT OF INTEREST STATEMENT

The authors declare that there are no conflicts of interest regarding the publication of this article.

## CONSENT STATEMENT

All authors gave consent for manuscript publication.

## Supporting information


Appendix S1.

## Data Availability

The data sets used and/or analysed during the current study are available from the corresponding author on request.

## References

[1] Benjamin EJ , Muntner P , Alonso A , et al. Heart disease and stroke Statistics‐2019 update: a report from the American Heart Association. Circulation. 2019;139:e56‐e528. 30700139 10.1161/CIR.0000000000000659

[2] Nakamura M , Sadoshima J . Mechanisms of physiological and pathological cardiac hypertrophy. Nat Rev Cardiol. 2018;15:387‐407. 29674714 10.1038/s41569-018-0007-y

[3] Niemi H , Honkonen K , Korpisalo P , et al. HIF‐1α and HIF‐2α induce angiogenesis and improve muscle energy recovery. Eur J Clin Invest. 2014;44:989‐999. 25208310 10.1111/eci.12333

[4] Aubert G , Martin OJ , Horton JL , et al. The failing heart relies on ketone bodies as a fuel. Circulation. 2016;133:698‐705. 26819376 10.1161/CIRCULATIONAHA.115.017355 PMC4766035

[5] Bedi KC Jr , Snyder NW , Brandimarto J , et al. Evidence for intramyocardial disruption of lipid metabolism and increased myocardial ketone utilization in advanced human heart failure. Circulation. 2016;133:706‐716. 26819374 10.1161/CIRCULATIONAHA.115.017545 PMC4779339

[6] Duncan JG , Finck BN . The PPARalpha‐PGC‐1alpha Axis controls cardiac energy metabolism in healthy and diseased myocardium. PPAR Res. 2008;2008:253817. 18288281 10.1155/2008/253817 PMC2225461

[7] Arany Z , Novikov M , Chin S , Ma Y , Rosenzweig A , Spiegelman BM . Transverse aortic constriction leads to accelerated heart failure in mice lacking PPAR‐gamma coactivator 1alpha. Proc Natl Acad Sci U S A. 2006;103:10086‐10091. 16775082 10.1073/pnas.0603615103 PMC1502510

[8] Bugger H , Schwarzer M , Chen D , et al. Proteomic remodelling of mitochondrial oxidative pathways in pressure overload‐induced heart failure. Cardiovasc Res. 2010;85:376‐384. 19843514 10.1093/cvr/cvp344

[9] Oka S , Alcendor R , Zhai P , et al. PPARα‐Sirt1 complex mediates cardiac hypertrophy and failure through suppression of the ERR transcriptional pathway. Cell Metab. 2011;14:598‐611. 22055503 10.1016/j.cmet.2011.10.001 PMC3217210

[10] Oka S , Zhai P , Yamamoto T , et al. Peroxisome proliferator activated receptor‐α association with silent information regulator 1 suppresses cardiac fatty acid metabolism in the failing heart. Circ Heart Fail. 2015;8:1123‐1132. 26443578 10.1161/CIRCHEARTFAILURE.115.002216 PMC4651813

[11] Warren JS , Oka S‐I , Zablocki D , Sadoshima J . Metabolic reprogramming via PPARα signaling in cardiac hypertrophy and failure: from metabolomics to epigenetics. Am J Physiol Heart Circ Physiol. 2017;313:H584‐H596. 28646024 10.1152/ajpheart.00103.2017 PMC6425516

[12] Ponikowski P , Voors AA , Anker SD , et al. 2016 ESC guidelines for the diagnosis and treatment of acute and chronic heart failure: the task force for the diagnosis and treatment of acute and chronic heart failure of the European Society of Cardiology (ESC) developed with the special contribution of. Eur Heart J. 2016;37:2129‐2200. 27206819 10.1093/eurheartj/ehw128

[13] Comitê Coordenador da Diretriz de Insuficiência Cardíaca , Rohde LEP , Montera MW , et al. Diretriz Brasileira de Insuficiência Cardíaca Crônica e Aguda. Arq Bras Cardiol. 2018;111:436‐539. doi:10.5935/abc.20180190 30379264

[14] Souza RWA , Piedade WP , Soares LC , et al. Aerobic exercise training prevents heart failure‐induced skeletal muscle atrophy by anti‐catabolic, but not anabolic actions. PloS One. 2014;9:e110020. 25330387 10.1371/journal.pone.0110020 PMC4201522

[15] de Souza PAT , de Souza RW , Soares LC , et al. Aerobic training attenuates nicotinic acethylcholine receptor changes in the diaphragm muscle during heart failure. Histol Histopathol. 2015;30:801‐811. 25548098 10.14670/HH-11-581

[16] Mota GAF , de Souza SLB , da Silva VL , et al. Cardioprotection generated by aerobic exercise training is not related to the proliferation of cardiomyocytes and angiotensin‐(1‐7) levels in the hearts of rats with Supravalvar aortic stenosis. Cell Physiol Biochem. 2020;54:719‐735. 32730701 10.33594/000000251

[17] Gomes MJ , Martinez PF , Campos DHS , et al. Beneficial effects of physical exercise on functional capacity and skeletal muscle oxidative stress in rats with aortic stenosis‐induced heart failure. Oxid Med Cell Longev. 2016;2016:8695716. 26904168 10.1155/2016/8695716 PMC4745811

[18] Reyes DRA , Gomes MJ , Rosa CM , et al. Exercise during transition from compensated left ventricular hypertrophy to heart failure in aortic stenosis rats. J Cell Mol Med. 2019;23:1235‐1245. 30456799 10.1111/jcmm.14025 PMC6349163

[19] Kolwicz SC . An “exercise” in cardiac metabolism. Front Cardiovasc Med. 2018;5:66. 29930946 10.3389/fcvm.2018.00066 PMC5999753

[20] Batista DF , Polegato BF , da Silva RC , et al. Impact of modality and intensity of early exercise training on ventricular remodeling after myocardial infarction. Oxid Med Cell Longev. 2020;2020:1‐6. 10.1155/2020/5041791 PMC7387991 32765807

[21] Brown MD . Exercise and coronary vascular remodelling in the healthy heart. Exp Physiol. 2003;88:645‐658. 12955165 10.1113/eph8802618

[22] Bernardo BC , McMullen JR . Molecular aspects of exercise‐induced cardiac remodeling. Cardiol Clin. 2016;34:515‐530. 27692221 10.1016/j.ccl.2016.06.002

[23] Holloway TM , Bloemberg D , da Silva ML , Simpson JA , Quadrilatero J , Spriet LL . High intensity interval and endurance training have opposing effects on markers of heart failure and cardiac remodeling in hypertensive rats. PloS One. 2015;10:e0121138. 25803693 10.1371/journal.pone.0121138 PMC4372563

[24] Moraes‐Teixeira Jde A , Félix A , Fernandes‐Santos C , et al. Exercise training enhances elastin, fibrillin and nitric oxide in the aorta wall of spontaneously hypertensive rats. Exp Mol Pathol. 2010;89:351‐357. 20800592 10.1016/j.yexmp.2010.08.004

[25] White FC , Bloor CM , McKirnan MD , Carroll SM . Exercise training in swine promotes growth of arteriolar bed and capillary angiogenesis in heart. J Appl Physiol. 1998;85:1160‐1168. 9729595 10.1152/jappl.1998.85.3.1160

[26] Bregagnollo EA , Zornoff LAM , Okoshi K , et al. Myocardial contractile dysfunction contributes to the development of heart failure in rats with aortic stenosis. Int J Cardiol. 2007;117:109‐114. 16839627 10.1016/j.ijcard.2006.06.006

[27] Wisløff U , Støylen A , Loennechen JP , et al. Superior cardiovascular effect of aerobic interval training versus moderate continuous training in heart failure patients: a randomized study. Circulation. 2007;115:3086‐3094. 17548726 10.1161/CIRCULATIONAHA.106.675041

[28] de Souza SLB , Ferreira Mota GA , da Silva VL , et al. Adjustments in β‐adrenergic signaling contribute to the amelioration of cardiac dysfunction by exercise training in Supravalvular aortic stenosis. Cell Physiol Biochem. 2020;54:665‐681. 32639114 10.33594/000000247

[29] de Souza SLB , Mota GAF , Gregolin CS , et al. Exercise training attenuates cirrhotic cardiomyopathy. J Cardiovasc Transl Res. 2020;14:674‐684. doi:10.1007/s12265-020-09997-0 32246321

[30] Hsia CCW , Hyde DM , Ochs M , Weibel ER . An official research policy statement of the American Thoracic Society/European Respiratory Society: standards for quantitative assessment of lung structure. Am J Respir Crit Care Med. 2010;181:394‐418. 20130146 10.1164/rccm.200809-1522ST PMC5455840

[31] Bieber LL , Abraham T , Helmrath T . A rapid spectrophotometric assay for carnitine palmitoyltransferase. Anal Biochem. 1972;50:509‐518. 4630394 10.1016/0003-2697(72)90061-9

[32] Vileigas DF , de Souza SLB , Corrêa CR , et al. The effects of two types of Western diet on the induction of metabolic syndrome and cardiac remodeling in obese rats. J Nutr Biochem. 2021;92:108625. 33705955 10.1016/j.jnutbio.2021.108625

[33] Pagan LU , Damatto RL , Cezar MDM , et al. Long‐term low intensity physical exercise attenuates heart failure development in aging spontaneously hypertensive rats. Cell Physiol Biochem. 2015;36:61‐74. 25924734 10.1159/000374053

[34] Sant'Ana PG , Batah SS , Leão PS , et al. Heart remodeling produced by aortic stenosis promotes cardiomyocyte apoptosis mediated by collagen V imbalance. Pathophysiology. 2018;25:373‐379. doi:10.1016/j.pathophys.2018.07.001 30030016

[35] de Brito Vieira WH , Ferraresi C , Schwantes MLB , et al. Photobiomodulation increases mitochondrial citrate synthase activity in rats submitted to aerobic training. Lasers Med Sci. 2018;33:803‐810. 29280079 10.1007/s10103-017-2424-2

[36] Pagan LU , Gomes MJ , Damatto RL , et al. Aerobic exercise during advance stage of uncontrolled arterial hypertension. Front Physiol. 2021;12:675778. 34149455 10.3389/fphys.2021.675778 PMC8209380

[37] Silva MG , Nunes P , Oliveira P , et al. Long‐term aerobic training improves mitochondrial and antioxidant function in the liver of Wistar rats preventing hepatic age‐related function decline. Biology (Basel). 2022;11:1750. 36552260 10.3390/biology11121750 PMC9774900

[38] Shangguan R , Hu Z , Luo Y , et al. Intramuscular mitochondrial and lipid metabolic changes of rats after regular high‐intensity interval training (HIIT) of different training periods. Mol Biol Rep. 2023;50:2591‐2601. 36626064 10.1007/s11033-022-08205-3

[39] Hein S , Arnon E , Kostin S , et al. Progression from compensated hypertrophy to failure in the pressure‐overloaded human heart. Circulation. 2003;107:984‐991. 12600911 10.1161/01.cir.0000051865.66123.b7

[40] Oka T , Akazawa H , Naito AT , Komuro I . Angiogenesis and cardiac hypertrophy: maintenance of cardiac function and causative roles in heart failure. Circ Res. 2014;114:565‐571. 24481846 10.1161/CIRCRESAHA.114.300507

[41] Gogiraju R , Xu X , Bochenek ML , et al. Endothelial p53 deletion improves angiogenesis and prevents cardiac fibrosis and heart failure induced by pressure overload in mice. J Am Heart Assoc. 2015;4:e001770. 25713289 10.1161/JAHA.115.001770 PMC4345879

[42] Shiojima I . Disruption of coordinated cardiac hypertrophy and angiogenesis contributes to the transition to heart failure. J Clin Invest. 2005;115:2108‐2118. 16075055 10.1172/JCI24682 PMC1180541

[43] Izumiya Y , Shiojima I , Sato K , et al. Vascular endothelial growth factor blockade promotes the transition from compensatory cardiac hypertrophy to failure in response to pressure overload. Hypertension. 2006;47:887‐893. 16567591 10.1161/01.HYP.0000215207.54689.31 PMC3132898

[44] Campos DH , Leopoldo AS , Lima‐Leopoldo AP , et al. Obesity preserves myocardial function during blockade of the glycolytic pathway. Arq Bras Cardiol. 2014;103:330‐337. 25352507 10.5935/abc.20140135 PMC4206364

[45] Miyachi M , Yazawa H , Furukawa M , et al. Exercise training alters left ventricular geometry and attenuates heart failure in dahl salt‐sensitive hypertensive rats. Hypertension. 2009;53:701‐707. 19255362 10.1161/HYPERTENSIONAHA.108.127290

[46] Weeks KL , Gao X , du XJ , et al. Phosphoinositide 3‐kinase p110α is a master regulator of exercise‐induced cardioprotection and PI3K gene therapy rescues cardiac dysfunction. Circ Heart Fail. 2012;5:523‐534. 22705768 10.1161/CIRCHEARTFAILURE.112.966622

[47] Sant'Ana PG , Tomasi LC , Murata GM , et al. Hypoxia‐inducible factor 1‐alpha and glucose metabolism during cardiac remodeling progression from hypertrophy to heart failure. Int J Mol Sci. 2023;24:6201. 37047174 10.3390/ijms24076201 PMC10094437

[48] Doenst T , Pytel G , Schrepper A , et al. Decreased rates of substrate oxidation ex vivo predict the onset of heart failure and contractile dysfunction in rats with pressure overload. Cardiovasc Res. 2010;86:461‐470. 20035032 10.1093/cvr/cvp414

[49] Barger PM , Kelly DP . Fatty acid utilization in the hypertrophied and failing heart: molecular regulatory mechanisms. Am J Med Sci. 1999;318:36‐42. 10408759 10.1097/00000441-199907000-00006

[50] Casquel De Tomasi L , Salomé Campos DH , Grippa Sant'Ana P , et al. Pathological hypertrophy and cardiac dysfunction are linked to aberrant endogenous unsaturated fatty acid metabolism. PloS One. 2018;13:e0193553. 29494668 10.1371/journal.pone.0193553 PMC5832311

[51] Doenst T , Nguyen TD , Abel ED . Cardiac metabolism in heart failure. Circ Res. 2013;113:709‐724. 23989714 10.1161/CIRCRESAHA.113.300376 PMC3896379

[52] Dobrzyn P , Pyrkowska A , Duda MK , et al. Expression of lipogenic genes is upregulated in the heart with exercise training‐induced but not pressure overload‐induced left ventricular hypertrophy. Am J Physiol Endocrinol Metab. 2013;304:E1348‐E1358. 23632628 10.1152/ajpendo.00603.2012

[53] Strøm CC , Aplin M , Ploug T , et al. Expression profiling reveals differences in metabolic gene expression between exercise‐induced cardiac effects and maladaptive cardiac hypertrophy. FEBS J. 2005;272:2684‐2695. 15943803 10.1111/j.1742-4658.2005.04684.x

[54] Stølen T , Shi M , Wohlwend M , et al. Effect of exercise training on cardiac metabolism in rats with heart failure. Scand Cardiovasc J. 2020;54:84‐91. 31500456 10.1080/14017431.2019.1658893

[55] Lachance D , Dhahri W , Drolet MC , et al. Endurance training or beta‐blockade can partially block the energy metabolism remodeling taking place in experimental chronic left ventricle volume overload. BMC Cardiovasc Disord. 2014;14:190. 25518920 10.1186/1471-2261-14-190 PMC4279960

[56] Kinney LaPier TL , Rodnick KJ . Effects of aerobic exercise on energy metabolism in the hypertensive rat heart. Phys Ther. 2001;81:1006‐1017. 11276183

[57] Osorio JC , Stanley WC , Linke A , et al. Impaired myocardial fatty acid oxidation and reduced protein expression of retinoid X receptor‐α in pacing‐induced heart failure. Circulation. 2002;106:606‐612. 12147544 10.1161/01.cir.0000023531.22727.c1

[58] Smeets PJH , de Vogel‐van den Bosch HM , Willemsen PHM , et al. Transcriptomic analysis of PPARα‐dependent alterations during cardiac hypertrophy. Physiol Genomics. 2008;36:15‐23. 18812456 10.1152/physiolgenomics.90296.2008

[59] Ingwall JS . On the hypothesis that the failing heart is energy starved: lessons learned from the metabolism of ATP and creatine. Curr Hypertens Rep. 2006;8:457‐464. 17087856 10.1007/s11906-006-0023-x

[60] Neubauer S . The failing heart — an engine out of fuel. N Engl J Med. 2007;356:1140‐1151. 17360992 10.1056/NEJMra063052

