# Supplementary material for: Effects of early exercise on cardiac function and lipid metabolism pathway in heart failure
Source: J Cell Mol Med. 2023 Aug 31;27(19):2956–69. doi: 10.1111/jcmm.17908 (PMC10538274; doi:10.1111/jcmm.17908)
Supplement: Supplementary file 1 — Appendix S1. [file JCMM-27-2956-s001.docx]

**SUPPLEMENTARY INFORMATION**


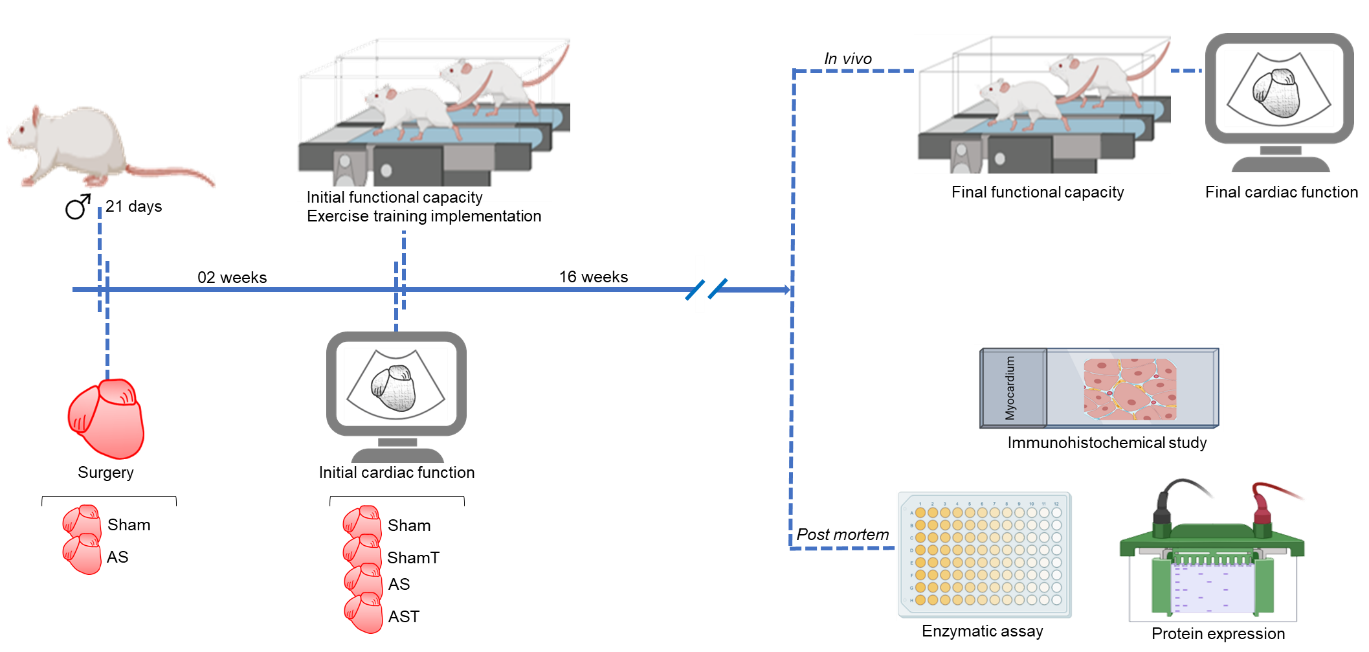


**Supplementary Figure 1.** Schematic representation of the experimental design. Sham and ShamT, untrained and trained control groups respectively; AS and AST, untrained and trained aortic stenosis groups.

**Supplementary Table 1.** Nutritional profile

|  | Sham | Sham-T | AS | AS-T |
| --- | --- | --- | --- | --- |
| **Boddy weight (g)** |  |  |  |  |
| Initial | 77.1 ± 14.3 | 82.8 ± 16.4 | 80.3 ± 9.76 | 72.0 ± 18.7 |
| Final | 492 ± 64.8 | 430 ± 58.9* | 420 ± 47.9* | 368 ± 49.9^&#^ |
| Fat deposits (g) |  |  |  |  |
| **Epididymal** | 4.09 ± 1.45 | 5.07 ± 1.85 | 2.70 ± 1.63 | 2.75 ± 0.73^&^ |
| Retroperitoneal | 6.57 ± 2.61 | 6.28 ± 1.78 | 4.06 ± 2.31* | 4.03 ± 0.87^&^ |
| Visceral | 4.07 ± 1.26 | 4.12 ± 0.84 | 2.33 ± 1.48* | 2.44 ± 0.46^&^ |
| Total | 14.7 ± 4.86 | 15.5 ± 3.77 | 9.10 ± 4.98* | 9.22 ± 1.40^&^ |
| **Daily food intake (g)** |  |  |  |  |
| Initial | 16.8 ± 2.96 | 18.1 ± 7.71 | 16.0 ± 6.34 | 18.0 ± 5.93 |
| Final | 32.5 ± 3.24 | 35.6 ± 2.65 | 25.0 ± 1.65* | 30.4 ± 3.39^&#^ |

ShamT, trained Sham; AS, untrained aortic stenosis; AST, trained aortic stenosis. Data expressed as mean ± SD. p < 0.05. * *vs*. Sham; ^&^ vs ShamT; ^#^ *vs*. AS. (n = 14-16 each group).

**Supplementary Table 2.** Cardiac anatomical data

|  | Sham | ShamT | AS | AST |
| --- | --- | --- | --- | --- |
| Tibia (cm) | 4.41 ± 0.08 | 4.28 ± 0.08 | 4.27 ± 0.12 | 4.19 ± 0.17 |
| Heart (g) | 1.18 ± 0.11 | 1.11 ± 0.18 | 2.47 ± 0.29* | 1.81 ± 0.29^&#^ |
| LV (g) | 0.82 ± 0.08 | 0.78 ± 0.13 | 1.50 ± 0.16* | 1.26 ± 0.16^&#^ |
| RV (g) | 0.24 ± 0.02 | 0.23 ± 0.04 | 0.53 ± 0.08* | 0.32 ± 0.08^&#^ |
| Atria (g) | 0.11 ± 0.01 | 0.11 ± 0.01 | 0.44 ± 0.09* | 0.27 ± 0.09^&#^ |
| Heart/Tibia | 0.27 ± 0.02 | 0.26 ± 0.04 | 0.58 ± 0.06* | 0.44 ± 0.07^&#^ |
| LV/Tibia | 0.19 ± 0.02 | 0.18 ± 0.03 | 0.35 ± 0.03* | 0.30 ± 0.04^&#^ |
| RV/Tibia | 0.05 ± 0.005 | 0.05 ± 0.008 | 0.12 ± 0.01* | 0.07 ± 0.02^&#^ |
| Atria/Tibia | 0.03 ± 0.003 | 0.03 ± 0.004 | 0.10 ± 0.02* | 0.06 ± 0.02^&#^ |

ShamT, trained Sham; AS, untrained aortic stenosis; AST, trained aortic stenosis. LV, left ventricle; RV, right ventricle. Data are mean ± SD. p < 0.05. * *vs*. Sham; ^&^ vs ShamT; ^#^ *vs*. AS. (n = 14-16 each group).

**Supplementary Table 3. Initial echocardiogram (2th-week post-surgery)**

|  | Sham | Sham-T | AS | AS-T |
| --- | --- | --- | --- | --- |
| HR (bpm) | 389 ± 59.7 | 396 ± 59.5 | 434 ± 56.8 | 395 ± 64.3 |
| LVDD (mm) | 6.02 ± 0.38 | 5.91 ± 0.53 | 5.23 ± 0,52 | 5.72 ± 0.88 |
| LVSD (mm) | 2.30 ± 0.52 | 2.18 ± 0.09 | 2.10 ± 0.23 | 2.13 ± 0.47 |
| AO (mm) | 2.96 ± 0.13 | 3.00 ± 0.22 | 3.25 ± 0.23 | 3.02 ± 0.12 |
| LA (mm) | 3.29 ± 0.47 | 3.40 ± 0.40 | 3.87 ± 0.21^*^ | 4.16 ± 0.54^&^ |
| LA/AO (mm/g) | 1.11 ± 0.14 | 1.13 ± 0.10 | 1.19 ± 0.07 | 1.37 ± 0.15^&#^ |
| RWT (%) | 0.44 ± 0.03 | 0.46 ± 0.07 | 0.71 ± 0.13^†^ | 0.62 ± 0.09^&^ |
| MFS (%) | 29.7 ± 2.33 | 31.2 ± 4.49 | 25.7 ± 3,41 | 28.6 ± 3.36 |
| EFS (%) | 62.1 ± 6.06 | 62.8 ± 2.92 | 59.9 ± 1.39 | 62.9 ± 5.02 |
| PWSV (ms) | 80.0 ± 4,55 | 78.0 ± 8.55 | 71.1 ± 5.61 | 72.0 ± 6.83 |
| Tei index | 0.26 ± 0.07 | 0.24 ± 0.09 | 0.25 ± 0.08 | 0.25 ± 0.07 |
| TDI S (average, cm/s) | 5.25 ± 0.10 | 5.60 ± 0.45 | 5.51 ± 0.46 | 5.74 ± 0.45 |
| TRIV (ms) | 20.2 ± 3.50 | 21.8 ± 1.98 | 19.4 ± 4.28 | 21.4 ± 2.19 |
| EDT (ms) | 42.2 ± 3.77 | 47.4 ± 5.48 | 42.9 ± 7.99 | 44.8 ± 0.83 |
| Mitral E (cm/s) | 103 ± 13.8 | 107± 6.82 | 108 ± 12.9 | 107 ± 11.6 |
| Mitral A | 69.8 ± 20.0 | 72.3 ± 7.58 | 74.8 ± 13.4 | 78.0 ± 19.6 |
| E/A (cm/s) | 1.53 ± 0.26 | 1.48 ± 0,11 | 1.45 ± 0.11 | 1.43 ± 0.29 |
| TDI E’ (average, cm/s) | 5.85 ± 0.68 | 6.44 ± 1.43 | 5.75 ± 0.32 | 6.86 ± 0.51 |
| TDI A’ (average, cm/s) | 3.75 ± 0.52 | 4.36 ± 0.43 | 5.41 ± 1.60 | 4.34 ± 0.79 |
| E/E’ (cm/s) | 17.6 ± 1.06 | 17.5 ± 5.02 | 18.7 ± 2.06 | 15.6 ± 1.00 |
| E’/A’ (cm/s) | 1.57 ± 0.07 | 1.50 ± 0.38 | 1.32 ± 0.42 | 1.61 ± 0.21 |

ShamT, trained Sham; AS, untrained aortic stenosis; AST, trained aortic stenosis; HR, heart rate; LVDD and LVSD, left ventricular (LV) diastolic and systolic diameters; PWDT, posterior wall diastolic thickness; AO, aorta; LA, left atrial diameter; RWT, relative wall thickness; MFS, Mesocardial fractional shortening; EFS, endocardial fractional shortening; PWSV, posterior wall shortening velocity; Tei index, myocardial performance index; TDI S, tissue doppler imaging (TDI) systolic velocity of mitral annulus; IVRT, isovolumetric relaxation time; EDT, E-wave deceleration time; E/A, ratio between early (E) to late (A) diastolic mitral inflow; TDI E’ and A’, TDI of early (E’) and late (A’) diastolic velocity of mitral annulus. Data are mean ± SD or median and interquartiles. p < 0.05. * *vs*. Sham; ^&^ vs ShamT; ^#^ *vs*. AS. (n = 14-16 each group).

| Adaptation period  (days) | Intensity  (m/min) | Time  (min) |
| --- | --- | --- |
| 1º | 5 | 10 |
| 2º | 5 | 15 |
| 3º | 5 | 20 |
| 4º | 5 | 25 |
| 5º | 5 | 30 |
| Treadmill exercise test 1 | | |
| Exercise Training Program | | |
| Weeks | Intensity  (% ES) | Time  (min) |
| 1ª | 60 | 30 |
| 2ª | 60 | 40 |
| 3ª | 60 | 50 |
| 4ª | 60 | 60 |
| Treadmill exercise testing 2 | | |
| 5ª | 60 | 60 |
| 6ª | 60 | 60 |
| 7ª | 60 | 60 |
| 8ª | 60 | 60 |
| Treadmill exercise testing 3 | | |
| 9ª | 60 | 60 |
| 10ª | 60 | 60 |
| 11ª | 60 | 60 |
| 12ª | 60 | 60 |
| Treadmill exercise testing 4 | | |
| 13ª | 60 | 60 |
| 14ª | 60 | 60 |
| 15ª | 60 | 60 |
| 16ª | 60 | 60 |
| Final treadmill exercise test | | |

**Supplementary Square 1. Training exercise protocol.** ES, exhaustion speed achieved during treadmill exercise testing.
